# Characterization of bone marrow heterogeneity in NK-AML (M4/M5) based on single-cell RNA sequencing

**DOI:** 10.1186/s40164-023-00391-5

**Published:** 2023-03-06

**Authors:** Wenqi Wu, Zeyan Shi, Zhongyuan Tang, Huiqun Li, Xiaoke Huang, Xiaolin Liang, Jing Li, Yibin Yao, Weihua Zhao, Meiqing Wu, Jun Luo, Zhenfang Liu

**Affiliations:** Department of Hematology, The First Affiliated Hospital of Guangxi Medical University, Nanning, Guangxi China

**Keywords:** Single-cell RNA sequencing, Normal karyotype acute myeloid leukemia, Heterogeneity, Leukemia stem cell

## Abstract

Normal karyotype acute myeloid leukemia (NK-AML) is a heterogeneous hematological malignancy that contains a minor population of self-renewing leukemia stem cells (LSCs), which complicate efforts to achieve long-term survival. We performed single-cell RNA sequencing to profile 39,288 cells from 6 bone marrow (BM) aspirates including 5 NK-AML (M4/M5) patients and 1 healthy donor. The single-cell transcriptome atlas and gene expression characteristics of each cell population in NK-AML (M4/M5) and healthy BM were obtained. In addition, we identified a distinct LSC-like cluster with possible biomarkers in NK-AML (M4/M5) and verified 6 genes using qRT‒PCR and bioinformatic analyses. In conclusion, we utilized single-cell technologies to provide an atlas of NK-AML (M4/M5) cell heterogeneity, composition, and biomarkers with implications for precision medicine and targeted therapies.

**Supplementary Information:**

The online version contains supplementary material available at 10.1186/s40164-023-00391-5.

## Introduction

Normal karyotype acute myeloid leukemia (NK-AML) is a complex heterogenous disease, and its pathogenesis, disease evolution and prognosis have not been fully elucidated. NK-AML is the most common cytogenetic type of AML, accounting for 40–49% of adult AML and 20–25% of pediatric AML diagnoses [1, 2]. In NK-AML, Bullinger et al. first distinguished two distinct groups: the FAB M1/M2 subtype with FLT3 mutation, in which the GATA2, NOTCH1, DNMT3A and DNMT3B genes are highly expressed, and the FAB M4/M5 subtype, in which the abnormally expressed genes are associated with granulocyte and monocyte differentiation, the immune response, and hematopoietic stem cell survival. Of note, patients classified into these groups have different outcomes [3]. Leukemia stem cells (LSCs) are a minor fraction of self-renewing cells that are capable of initiating and maintaining leukemia [4]. AML LSCs have been demonstrated to exhibit self-renewal, relative quiescence, apoptosis resistance, and increased drug efflux, which likely render these cells less susceptible to conventional therapies aimed at bulk proliferative disease [5]. Therefore, for the purpose of eradicating AML and achieving long-term remission, treatment courses must eliminate the LSC population [6]. Recently, high-throughput sequencing has increased our knowledge of the genomic and transcriptome landscapes of AML [7, 8], but merging these data with the in vivo biology of LSCs is still in early stages. Thus, a powerful approach is needed to characterize malignant cell populations, such as LSCs, and their biological information.

In AML, traditional sequencing technologies have masked the characteristics of minor populations of leukemic cells, while single-cell sequencing can explore the differences in the genomic [9, 10], transcriptomic [11, 12] and epigenomic landscapes [13] of this disease between cells at single-cell resolution. The application of single-cell RNA sequencing (scRNA-seq) has led to the identification of new LSC populations and new markers in AML patients and has also identified independent factors of a poor prognosis and strategies to prevent AML recurrence [11, 12]. In AML, scRNA-seq identified intratumoral heterogeneity and distinguished malignant AML cells from normal cells. In brief, this technique provides a powerful means to potentially address questions related to stemness, developmental hierarchies, and interactions between malignant and immune cells [14]. However, the application of scRNA-seq in AML, the most common hematological malignant tumor in adults, is still in its infancy, especially in NK-AML, and there is no systematic description in the literature.

Here, we adapted scRNA-seq technology to acquire transcriptional data for thousands of single cells from bone marrow (BM) aspirates. We profiled 36,865 cells from 5 NK-AML (M4/M5) patients and 2423 cells from 1 healthy donor by scRNA-seq and acquired a total of 18 cell subpopulations. We also profiled the single-cell transcriptome atlas of BM cells from the NK-AML (M4/M5) patients and healthy donor. In addition, we revealed the existence of a key cell subset, which could be a group of LSC-like cells that may play key roles in the initiation and maintenance of NK-AML, as this population coexpressed multiple genes related to AML pathogenesis and a poor prognosis. Finally, through combination of clinical data from the GEO database with qRT‒PCR analysis results, we verified that integrin subunit α-4 (ITGA4), inositol 1,4,5-trisphosphate receptor type 2 (ITPR2), adhesion G protein-coupled receptor E2 (ADGRE2), ankyrin repeat domain 28 (ANKRD28), lysine demethylase 5B (KDM5B) and cyclin-dependent kinase 6 (CDK6) were significantly upregulated in NK-AML and that ITGA4 and ITPR2 may be biomarkers for predicting NK-AML prognosis.

## Methods

### Human specimen procurement and isolation

Five patients who were pathologically diagnosed with NK-AML (M4/M5) and one healthy volunteer at The First Affiliated Hospital of Guangxi Medical University between 2019 and 2020 were enrolled in this study. None of the patients were treated with chemotherapy, radiation or any other antitumor medicines prior to BM sample collection. This study was approved by the Ethics Committee of The First Affiliated Hospital of Guangxi Medical University. Written informed consent was obtained from every participant in accordance with the Declaration of Helsinki.

BM mononuclear cells were isolated using density gradient centrifugation according to the manufacturer’s instructions. In brief, 2 ml of fresh BM aspirate and 2 ml 1 × DPBS (Gibco) were collected in an EDTA anticoagulant tube and subsequently layered onto Lymphocyte Separation Medium. After centrifugation, BM mononuclear cells (the third layer) were carefully transferred to a new tube and washed with 1 × DPBS. After supernatant removal, the cell pellets were suspended in red blood cell lysis buffer (Solarbio) and incubated on ice for 10 min to lyse red blood cells. After washing twice with 1 × DPBS, the cell pellets were resuspended in cell freezing medium (90% fetal calf serum supplemented with 10% dimethyl sulfoxide (DMSO)). Finally, the BM mononuclear cells were viably frozen and stored in liquid nitrogen or a – 85 ℃ freezer.

Viably frozen cells were thawed using standard procedures. First, frozen cells were thawed at 37 °C, suspended in Dulbecco’s Modified Eagle Medium (DMEM) and washed with DMEM. The cell suspension was passed through a 40 µm filter after resuspension in DMEM at a concentration of 1–2 million cells per ml. Finally, we obtained a single-cell suspension. Cell counts and viability were determined with a hemocytometer with trypan blue staining (Gibco). Samples were analyzed on a Chromium system (10 × Genomics) according to the manufacturer’s instructions for an expected capture rate of 20,000 single cells per patient.

### Sample processing with the 10X genomics platform and cDNA library preparation

To process the previously mentioned single-cell suspensions, we added a single-cell sample, gel beads and partitioning oil to 10X Genomics Single Cell A Chip Kits and acquired gel beads in emulsion (GEMs). The GEMs were reverse transcribed using a Bio-Rad C1000 Touch. The conditions were as follows: 53 ℃ for 45 min, 85 ℃ for 5 min, and hold at 4 ℃. The hot cover was set to 53 ℃. After reverse transcription, cDNA was recovered using Recovery Agent, which was provided by 10X Genomics, and then purified with Silane DynaBeads as outlined in the user guide. Purified cDNA was amplified before being cleaned using SPRIselect beads to eliminate short fragments. The PCR conditions were as follows: 98 ℃ for 45 s; 98 ℃ for 20 s, 67 ℃ for 30 s, and 72 ℃ for 1 min for 12 cycles; and 72 ℃ for 1 min. The cDNA concentrations of samples were quantified using the Qubit3.0 Fluorometer (Invitrogen). The cDNA libraries were constructed using the Chromium Single Cell 5’ Library Kit. The constructed libraries were analyzed on an Illumina HiSeq2500 sequencer in the PE150 mode.

### scRNA-seq data processing

The Chromium-prepared sequencing data were demultiplexed and converted to FASTQ files with 10X Genomics Cell Ranger software (version 3.1.0). The same software package was used for filtering, alignment, and count quantification. The FASTQ files were aligned to the human reference genome GRCh38. These preliminary data were then analyzed with the R package Seurat. Cells with too many or too few genes or too much mitochondrial RNA were filtered out, as these might represent doublets. Specifically, cells with < 500 or > 4000 genes, a UMI ≥ 8000 or a mitochondrial gene percentage ≥ 10% were filtered. The expression value of each gene in a given cluster was compared against that in the rest of the cells using the Wilcoxon rank-sum test. Significantly upregulated genes were identified using the following criteria: (1) gene expression ≥ 1.28-fold in the target cluster, (2) gene expression of cells belonging to the target cluster > 25%, and (3) p value < 0.05. After rigorous quality control, a total of 39,288 cells and an average of approximately 2 × 10^4^ genes were retained in the six samples for subsequent scRNA-seq analysis.

After removing poor-quality cells from the dataset, we employed the global-scaling normalization method “LogNormalize” to normalize gene expression and identified highly variable genes in the single cells. Subsequently, the most variable genes were identified, and a linear dimensionality reduction approach (principal component analysis, PCA) was performed with the variable genes. The principal components were then included in a graph-based clustering algorithm. For visualization purposes, a nonlinear dimensionality reduction approach (t-distributed stochastic neighbor embedding, t-SNE) was used, and the t-SNE plots were colored according to the clusters determined in the previous step. Ultimately, we identified 18 clusters from the scRNA-seq data.

### TCGA and GTEx databases

We obtained BM mRNA expression data and clinical parameters from normal donors in the GTEx database and downloaded the mRNA expression data and clinical parameters of AML patients in the TCGA database. Then, we integrated and normalized the data from these two databases using the R package “GTEx.merge.R” and acquired differentially expressed genes (DEGs) by comparing the AML and normal control data. The R package “GTEx.Survival.R” was used to obtain prognosis-related genes and survival curves by survival analysis. P < 0.05 was considered statistically significant.

### Functional analysis

Genes with a P value < 0.01 and an absolute log2-fold change (log2FC) > 0.36 between a target cluster and other clusters were used for GO and KEGG pathway enrichment analyses. In addition, preranked gene set enrichment analysis (GSEA) was performed. The required input files were extracted from the expression matrix, and the enrichment analyses were performed using OmicShare tools. Interactions between DEGs were analyzed using the Gene Multiple Association Network Integration Algorithm (GeneMANIA; http://www.genemania.org/). The Search Tool for the Retrieval of Interacting Genes (STRING; https://string-db.org/) was used to investigate the protein‒protein interactions between DEGs.

### Survival analysis

The transcriptome data and clinical information of the GSE106291 dataset were downloaded from the GEO database. After normalizing the read counts and log2-transforming the gene expression values, the gene expression matrix of each patient was obtained. A median threshold of gene expression was used to categorize patients into high- and low-expression groups. A total of 250 AML patients and the top 76 DEGs from a cluster were selected for survival analysis to explore the relationship between the expression of target genes and patient clinical outcome. GraphPad Prism 7 software (GraphPad Software Inc., La Jolla, CA, USA) was used to visualize the Kaplan‒Meier estimates of survival curves. P < 0.05 was considered statistically significant.

### Quantitative real-time PCR (qRT-PCR)

A total of 30 BM samples were obtained from 20 chemotherapy-naive NK-AML (M4/M5) patients and 10 healthy volunteers between 2013 and 2016 at The First Affiliated Hospital of Guangxi Medical University. The NK-AML(M4/M5) patients were classified according to the French-American-British (FAB, 2016) Criteria. All NK-AML (M4/M5) patients received regular follow-up, and the follow-up period ended in December 2017. Patients who had other hematological diseases or malignant tumors were excluded. The healthy volunteers had no obvious abnormalities in any examination indexes. Written informed consent was obtained from all the participants according to the Declaration of Helsinki prior to BM collection. Detailed clinical features of the 30 samples are provided in Additional file 2: Table S1C.

Up to 2 ml of BM sample was extracted from each participant, and BM mononuclear cells were separated by density gradient centrifugation. Total RNA was isolated from the BM cells using TRIzol reagent (Invitrogen, USA) according to the manufacturer’s instructions. RNA was then reverse transcribed into cDNA. Reverse transcription was performed with a SuperScriptTM III Reverse Transcriptase kit (Invitrogen: 18080–044) on a Gene Amp PCR System 9700 (Applied Biosystems). qRT‒PCR was performed on a ViiA 7 Real-time PCR System (Applied Biosystems) using the 2X PCR Master Mix Kit (Arraystar). The following reaction conditions were used: 95 °C for 10 min, followed by 40 cycles of 95 °C for 10 s and 60 °C for 1 min. ACTB was used as an endogenous reference gene, and the primer sequences used in the present study were as follows: ACTB forward: 5′GTGGCCGAGGACTTTGATTG3′ and reverse: 5′CCTGTAACAACGCATCTCATATT3′; ITPR2 forward: 5′TGCGCCAATCAGCTACTTCT3′ and reverse: 5′TCAGGATTAAGCTCTGCAGCTA3′; ADGRE2 forward: 5′GGTCCTGGAACCTGAGAAGC3′ and reverse: 5′AGGTGCTGGTGTTCTGGATG3′; ANKRD28 forward: 5′TGGTCACCGTCTATGTCTTCAG3′ and reverse: 5′AGGGCTTATTGTTGCTCTATTATC3′; KDM5B forward: 5′AATAGAACCCGAGGAGACAACG3′ and reverse: 5′GACAGACATACAGGTCCACAGCA3′; ITGA4 forward: 5′CTGGGTAGCCCTAATGGA3′ and reverse: 5′ATGCCCACAAGTCACGAT3′; and CDK6 forward: 5′CATTCAAAATCTGCCCAACC3′ and reverse: 5′GGTCCTGGAAGTATGGGTGA3′. The relative expression of target genes was calculated with the comparative 2 −  ∆∆Ct method. As the data did not exhibit a normal distribution, the relative expression of target genes was compared among different groups using the Mann‒Whitney U test.

## Results

### Identification of cell populations in healthy BM samples

**Fig. 1:**
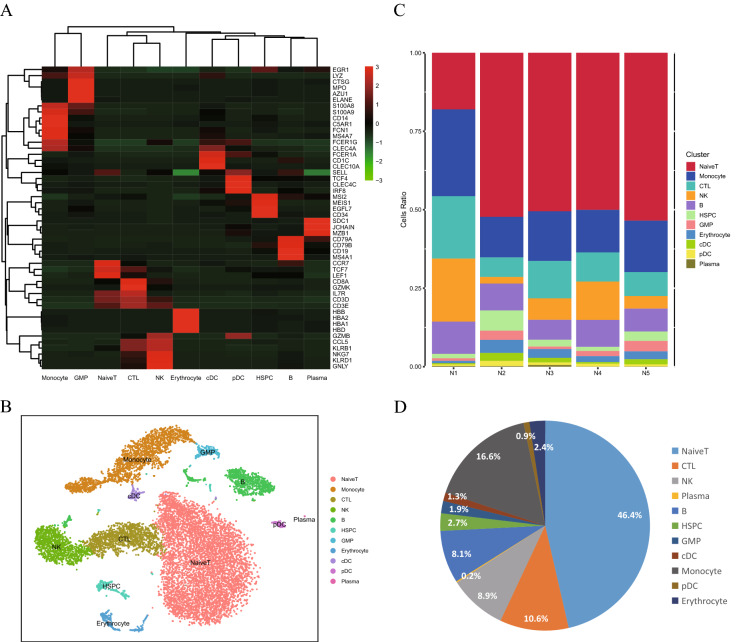
Dissection and clustering of the healthy BM sample. **A** t-SNE plot representation of BM cells from the normal control. **B** Proportional distribution of 5 main cell populations in BM cells from the normal control

To characterize the features and cellular diversity of healthy BM, we performed scRNA-seq using a single-cell suspension from a healthy donor on the high-throughput platform 10X Genomics Chromium. To obtain a more representative transcriptomic profile of healthy BM samples, we integrated four other healthy donors from the GEO database (GSM3396162, GSM3396167, GSM3396172, and GSM3396185) (Additional file 2: Table S1A). A total of 14,689 high quality cells were isolated and sequenced with a median of 2859 UMI counts and 904 genes detected per cell. We distinguished 15 clusters consisting of cells in the range of 30–6816 cells per cluster by unsupervised clustering analysis. Based on the marker genes of each cluster, such as CD3D/CD3E for T cells and CD19 for B cells (Fig. 1A), we merged the 15 clusters into 9 main cell populations, which corresponded to hematopoietic stem/progenitor cells (HSPCs), granulocyte–macrophage progenitors (GMPs), T cells, natural killer (NK) cells, B cells, plasma cells, monocytes, erythrocytes and dendritic cells (DCs) (Fig. 1B). Basically, the similar cell clusters gathered together in a range. Our cell type annotations were consistent with published gene signatures and researches. [14–16]. All 15 cell types were identified in all 5 samples (Fig. 1C). We then explored the distribution proportions of these clusters and found that T cells were dominant in the BM mononuclear cells of normal controls (more than 50%), followed by monocytes (approximately 20%) and B cells (approximately 10%), which was in accordance with previous results [17] (Fig. 1D). Thus, scRNA-seq analysis of normal BM revealed diverse hematopoietic cell types and suggested a distribution pattern consistent with current views on hematopoiesis.

### Single-cell profiling of NK-AML(M4/M5) tumor ecosystems

**Fig. 2:**
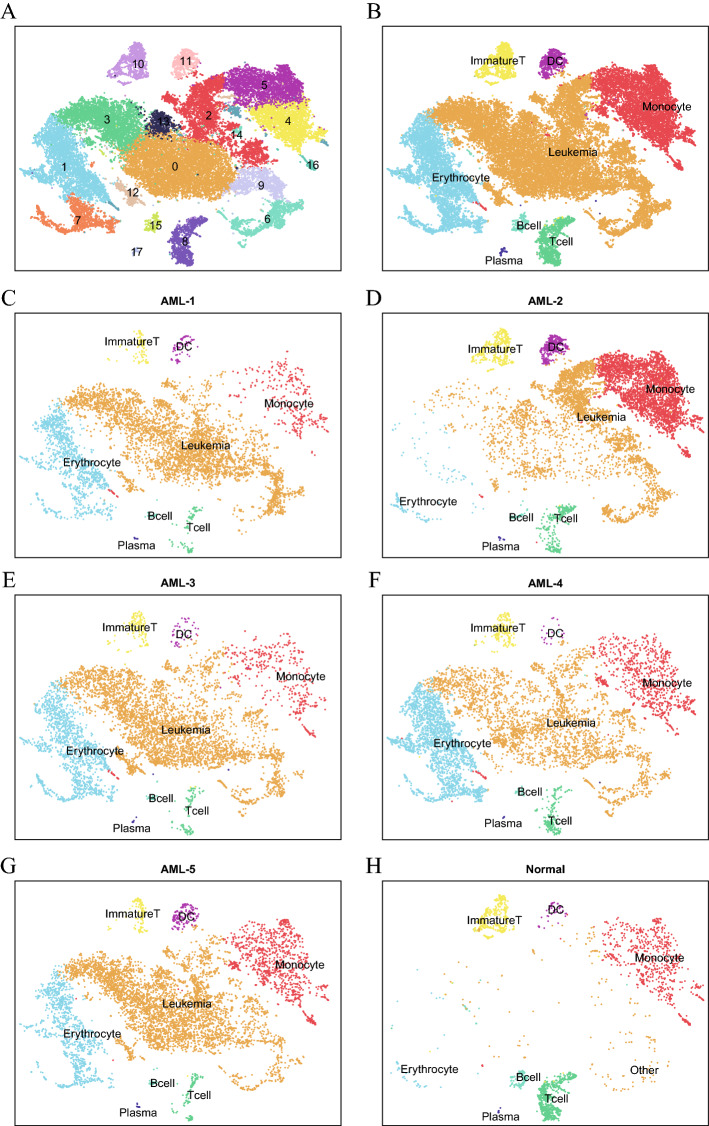
Dissection and clustering of BM cells in 5 NK-AML (M4/M5) samples and 1 healthy control sample. **A** t-SNE visualization showing the single-cell transcriptomes of all 18 clusters from 5 NK-AML (M4/M5) patients and 1 normal control. **B**–**H** t-SNE plot representation of 8 merged cell clusters from all 6 samples includingAML-1, AML-2, AML-3, AML-4, AML-5, and normal control. Each dot corresponds to a single cell, and clusters are colored distinctively according to cell type and location

To examine cellular diversity in NK-AML, we also carried out scRNA-seq with viably frozen BM cells from 5 treatment-naive NK-AML (M4/M5) patients without enrichment to achieve a broad overview of the transcriptional profile and gene expression patterns of NK-AML at the single-cell level. To compare the similarities and differences between NK-AML (M4/M5) patients and the normal control, we combined the data from 6 samples for further analysis. Based on the gene expression characteristics of individual cells, we applied Seurat software to identify cell clusters and visualized them after dimensionality reduction by t-SNE. After rigorous quality control, a total of 39,288 effective cells and an average of approximately 2 × 10^4^ genes per patient (range 19,316–20,409) were retained from the 6 samples for subsequent scRNA-seq analysis (Additional file 2: Table S1B). According to the DEGs and known cell type-specific markers of each cluster, we identified 7 clusters: erythrocytes (clusters 1 and 7), monocytes (clusters 4, 5, 14 and 16), T cells (including NK cells, cluster 8), immature T cells (cluster 10), DCs (cluster 11), B cells (cluster 15) and plasma cells (cluster 17). The top 5 DEGs of each cluster are presented in Table 1. In addition, the remaining clusters (clusters 0, 2, 3, 6, 9, 12 and 13), which lacked common markers for normal hematopoietic cells and were almost exclusively derived from the NK-AML (M4/M5) samples, were considered to be leukemic cells (Fig. 2A). This allowed us to merge the 18 clusters into 8 main cell populations (Fig. 2B). Among the NK-AML (M4/M5) patients, there were significant differences in cell population abundance even if the patients were of the same FAB type, which revealed tumor heterogeneity among the patients (Fig. 2C–G).

**Table 1:** The top 5 DEGs of 18 clusters

Cluster 0	Cluster 1	Cluster 2	Cluster 3	Cluster 4	Cluster 5	Cluster 6	Cluster 7	Cluster 8	Cluster 9	Cluster 10	Cluster 11	Cluster 12	Cluster 13	Cluster 14	Cluster 15	Cluster 16	Cluster 17
C1QTNF4	AC104232.1	ELANE	RHEX	CD300E	S100A12	UBE2C	AHSP	S1PR5	MCM10	MAL	CD1C	MIR181A1HG	MAMDC2	PTGS1	MS4A1	C1QA	IGHG1
AL590226.1	CNRIP1	CTSG	LINC02573	SLC7A7	PLBD1	GTSE1	APOC1	FGFBP2	CDC45	TCF7	PKIB	ZNF385D	TSPAN2	LMNA	LINC02397	FCGR3A	IGHG2
TRH	CYP7B1	AZU1	IKZF2	GPBAR1	S100A8	AURKB	KCNH2	FCRL6	CDC6	CCR7	ACY3	MIR222HG	AC007384.1	LAT	FCRL1	LINC02432	IGKV4-1
CD200	SLC40A1	HOXB-AS3	ZNF385D	MAFB	S100A9	TOP2A	ANK1	GZMB	GINS2	CAMK4	NDRG2	IKZF2	SPACA9	HEXIM1	FCRLA	ZNF703	SDC1
RAB7B	HPGD	RNASE2	MLLT11	CLEC7A	CDA	HMMR	HBB	KLRD1	UHRF1	IL7R	FCER1A	FTX	SPINK2	RAB27B	TCL1A	CALML4	IGHA1

When comparing the AML patients and normal donor, we observed that the number of cells in the NK-AML (M4/M5) samples was greater than that in the normal sample. With the normal control, we also obtained 8 clusters (Fig. 2H). However, there was significant heterogeneity in the cell proportions between the normal sample and NK-AML (M4/M5) samples. The normal control samples were mainly composed of monocytes, T cells and B cells, while there were more leukemic cells and fewer lymphocytes in the NK-AML (M4/M5) samples (Fig. 2C–H). These findings suggest the malignant clonal proliferation of leukemic cells in AML. The normal hematopoietic cells and leukemic cells clustered together, indicating that malignant cells may have an expression pattern similar to that of normal cells.

### Cluster 12 may be LSC-like cells

**Fig. 3:**
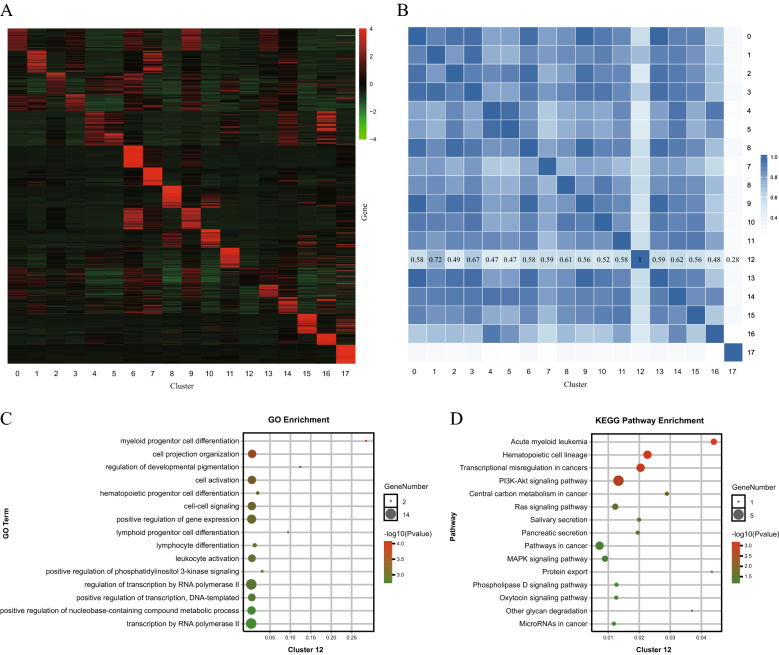
Identification gene expression pattern and gene function in 18 cell clusters. **A** Heatmap shows the expression of top 50 DEGs in 18 clusters. The number of genes was too large (757 genes) to present all genes names. (B) Pearson similarity analysis of gene expression matrix of each cell subpopulation. **C**–**D** GO and KEGG pathway enrichment analyses of cluster 12

To identify the similarities and differences in gene expression among the 18 cell subsets, the top 50 DEGs (757 genes in total) of each cluster were selected for heatmap analysis (Fig. 3A). The gene expression patterns of a single cell type (for example, clusters 4, 5, 14 and 16) were similar, consistent with the cell annotation results. Gene expression patterns varied widely among the cell types. Pearson correlation analysis was performed to more intuitively understand the similarities among cell subsets (Fig. 3B). The results demonstrated that cluster 12 showed low similarity to the other clusters apart from cluster 17, which was classified as plasma cells and was significantly different. In addition, from the t-SNE plot (Fig. 2A), we observed that a continuous arc was formed by the 12 myeloid cell clusters, and the 5 lymphoid cell clusters were distributed on both sides, which may indicate the differentiation process of hematopoietic cells. However, cluster 12 appeared to be separated from other clusters. These results all indicated that cluster 12 was obviously specific.

To predict the functions of DEGs in AML, GO and KEGG pathway enrichment analyses were performed for the top 50 DEGs of each cluster (Additional file 1: Figure S1). Cluster 1, which expressed the primary genes GATA1 and GATA2, exhibited enrichment in erythrocyte differentiation and homeostasis and was considered early erythrocytes. Cluster 7 was related to the heme metabolic process, the porphyrin-containing compound metabolic process and hemoglobin binding, indicating that it was involved in the synthesis, binding and transport of hemoglobin and comprised functionally mature erythrocytes. Monocytes (clusters 4, 5, 14 and 16) were involved in the immune and defense responses and immune cell activation. T cells (clusters 8 and 10) and B cells (clusters 15 and 17) were associated with the immune response and T/B-cell activation and proliferation. The genes in DCs (cluster 11) were enriched in the MHC protein complex, antigen processing and presentation. DCs are professional antigen-presenting cells that can efficiently take up, process and present antigens on MHC I and MHC II and effectively activate naive T cells. These events are central to initiating, regulating, and maintaining the immune response [18]. The data were consistent with our cell annotation results. Among leukemia cell populations, cluster 2 was related to the terms cytokine-cytokine receptor interaction and response to stimulus, defense and immune. Clusters 6 and 9 showed similar enrichment in the cell cycle process, chromosome organization and the p53 signaling pathway. It appeared that clusters 2, 6 and 9 were not sufficiently relevant to AML. Clusters 0, 3, 12 and 13 were related to transcriptional misregulation in cancer, acute myeloid leukemia, hematopoietic cell lineage, microRNAs in cancer and pathways in cancer (including the MAPK/PI3K-Akt/Rap1/p53/Ras/Phospholipase D signaling pathway). However, cluster 12 was also associated with myeloid/lymphoid progenitor cell differentiation, gene expression regulation and leukocyte activation, while clusters 0, 3 and 13 were enriched in cell proliferation, lymphocyte activation and differentiation, and cell secretion, respectively, by GO analysis. Cluster 12 was comprehensively and highly involved in AML-related signaling pathways (the PI3K-Akt/Ras/MAPK/Phospholipase D signaling pathway), acute myeloid leukemia, hematopoietic cell lineage, and transcriptional misregulation in cancers, suggesting that the function of cluster 12 was highly correlated with AML compared to that of the other leukemia cell clusters (Fig. 3C–D).

We further compared the surface markers of each cluster and found that the number of cells in cluster 12 accounted for only 0.51%-3.84% of the total but that these cells highly expressed CD34, CD38, CD96, CD46, CD34, CD47, CD82, CD44 and CD133, most of which are highly expressed in LSCs [19] (see Table 2). These markers were also expressed in the other 6 leukemia cell clusters. However, this pattern was not comprehensive, and the expression values of the other clusters were significantly lower than that of cluster 12. Cluster 12 was almost exclusively derived from the 5 NK-AML (M4/M5) samples (677 cells), and only a few cells (19 cells) came from the normal sample. The results showed that cluster 12 was more likely to be LSCs. In addition, the nontraditional LSC markers CD38 and CD46 were highly expressed in cluster 12. In conclusion, we hypothesized that cluster 12 was not traditional LSCs but might be a group of LSC-like cells that might play important roles in the initiation and maintenance of NK-AML.

**Table 2:** Expression of cell surface markers in each cell subpopulation

Cluster	Marker gene(log2FC)
0	CD34, CD133, CD200
1	CD36, CD55, CD63, CD82, CD84
2	None
3	CD7, CD38, CD82, CD244
4	CD1D, CD4, CD14, CD36, CD48, CD68, CD86, CD93, CD300A, CD300C, CD300E, CD302
5	CD14, CD36, CD48, CD68, CD86, CD93, CD300C, CD302
6	CD59
7	CD36, CD63, CD151, CD320
8	CD2, CD3D, CD3E, CD3G, CD7, CD8A, CD8B, CD47, CD48, CD52, CD53, CD69, CD247
9	CD59
10	CD2, CD3E, CD3D, CD3G, CD5, CD6, CD7, CD8A, CD8B, CD27, CD52, CD69, CD247
11	CD1C, CD1D, CD4, CD74, CD86, CD300C
12	CD34, CD38, CD44, CD46, CD47, CD82, CD96, CD99, CD133
13	CD44, CD99, CD133
14	CD14, CD36, CD68, CD84, CD86, CD151, CD300C
15	CD19, CD22, CD24, CD27, CD37, CD40, CD52, CD53, CD55, CD72, CD74, CD79A, CD79B, CD83, CD180
16	CD4, CD37, CD48, CD52, CD68, CD79B, CD86, CD300A, CD300C, CD300E
17	CD19, CD27, CD48, CD59, CD79A, CD79B, CD138, CD320

**Fig. 4:**
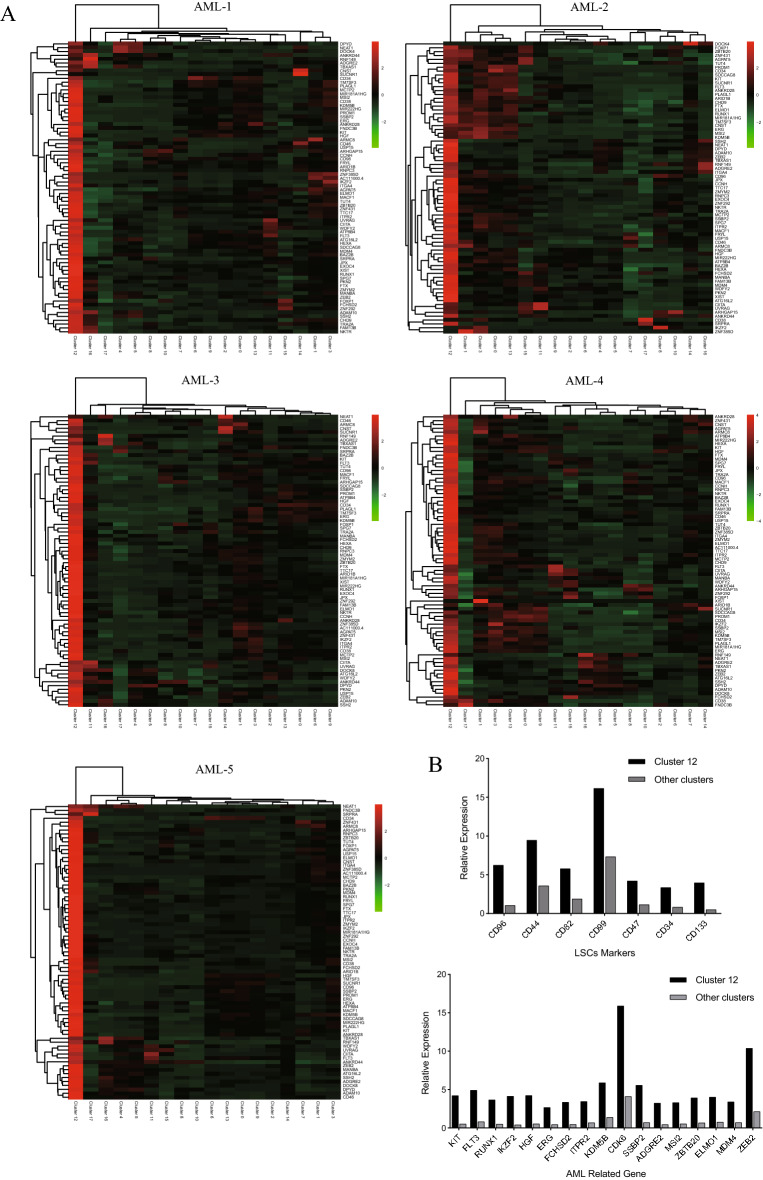
Expression discrepancy of the top 76 genes of interest between cluster 12 and other clusters. **A** Comparison of the expression levels of the 76 genes in cluster 12 with those in other clusters among NK-AML patients. **B** Relative expression of LSCs markers and AML-ralated genes from cluster 12

**Fig. 5:**
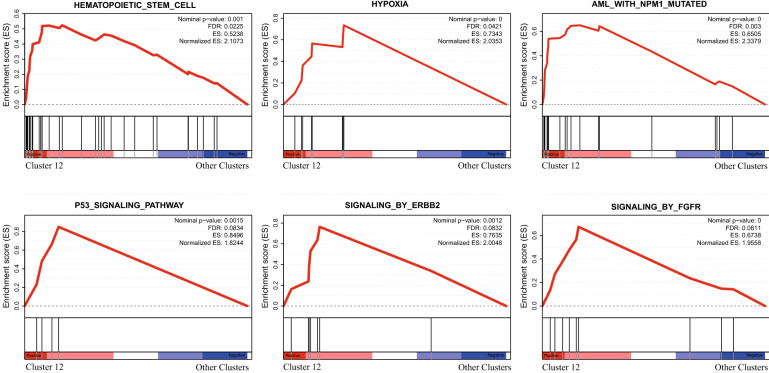
Representative GSEA plots of the top 76 differentially upregulated genes from cluster 12

To explore the gene expression pattern of cluster 12, a total of 76 upregulated genes were screened out from the cluster according to the gene expression cutoff of log2FC > 2 (Additional file 3: Table S2). There were significant differences in the abundance of cluster 12 among the NK-AML patients; thus, we compared the expression discrepancy of these 76 genes between cluster 12 and the other clusters in 5 AML samples respectively (Fig. 4A). The results showed that the expression levels of the 76 genes were all higher in cluster 12, indicating that the high expression characteristics were consistent in all NK-AML (M4/M5) samples. We observed that many of these genes, including LSC markers (CD96, CD34, CD47, CD82, CD44, CD99 and CD133) and AML-related genes (KIT, FLT3, RUNX1, IKZF2, HGF, SSBP2, FCHSD2, ADGRE2, ERG, MSI2, ZBTB20, ITPR2, ELMO1, MDM4, ZEB2, KDM5B, and CDK6), were associated with AML and were more highly expressed in cluster 12 than in the other clusters (Fig. 4B). Some of the genes were poor prognostic indicators in AML (such as CD133, KIT, HGF, ERG, FCHSD2, ADGRE2, ITPR2 and ELMO1). These findings showed that cluster 12 coexpressed multiple genes related to the pathogenesis and poor prognosis of AML. In addition, GSEA further confirmed the results of the GO and KEGG enrichment analyses. GSEA revealed significant activation of gene sets associated with hematopoietic stem cells, hypoxia, NPM1 mutation-related AML, p53 signaling pathways, and FGFR and ERBB2 receptor tyrosine kinase family-mediated signaling pathways in cluster 12 (Fig. 5). Thus, cluster 12 may be LSC-like cells, and the top 76 genes in this cluster were significantly related to the pathogenesis and poor prognosis of NK-AML.

### PCR analysis

**Fig. 6:**
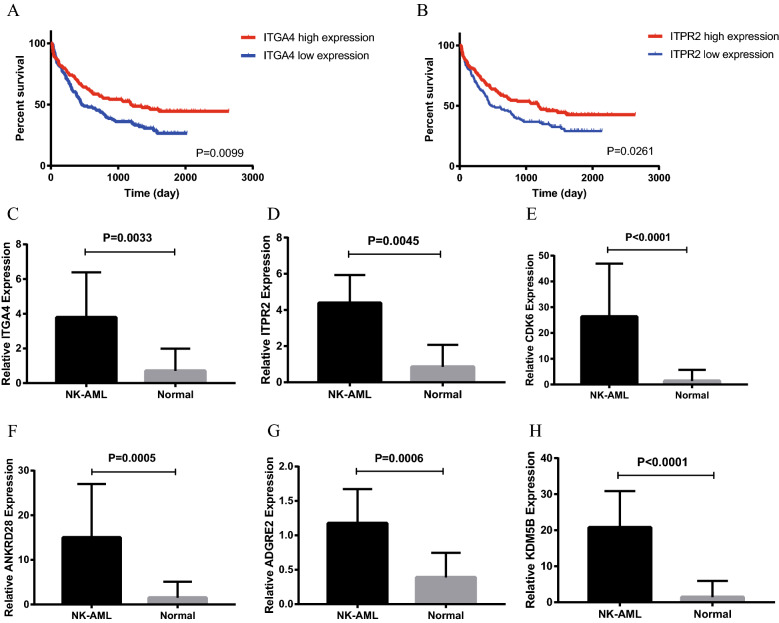
**A**–**B** Kaplan‒Meier survival curves of AML patients from the dataset GSE106291 (n = 250) using the ITGA4 and ITPR2 genes. **C**–**H** The relative expression of ITGA4, ITPR2, CDK6, ANKRD28, ADGRE2 and KDM5B in the NK-AML (M4/M5) group and the normal control group

To research the relationship between the expression of the 76 genes and prognosis in AML, we combined BM samples from AML patients in the TCGA database and those from normal controls in the GTEx database for analysis. Ultimately, 2800 differentially upregulated genes and 777 prognosis-related genes in AML were obtained. Venn diagram analysis showed that 29 of the differentially upregulated genes and 19 of the prognosis-related genes overlapped with the 76 genes identified with our data. Among them, 12 genes were upregulated in AML and associated with prognosis: MIR181A1HG, MIR222HG, KIT, HGF, MCTP2, FCHSD2, ERG, ITGA4, ITPR2, DPYD, CDK6, SPN, and ARHGEF6. Since MIR181A1HG and MIR222HG are RNA transcripts, we did not further analyze them. Afterward, the GSE106291 dataset, containing known treatment outcomes (including overall survival, OS) of 250 AML patients, was selected to determine the prognostic significance of the above 10 genes. Kaplan‒Meier analysis confirmed that the expression values of ITGA4 and ITPR2 could predict the OS outcomes of AML patients with P < 0.05, which was consistent with the results of the analysis of TCGA and GTEx data. Low expression levels of ITGA4 and ITPR2 indicated a poor outcome (Fig. 6A–B). We further conducted PCR validation and found that ITGA4 and ITPR2 were significantly upregulated in AML (Fig. 6C–D), but the sample size was too small for us to perform a significant survival analysis.

We next performed differential expression analysis between each NK-AML (M4/M5) patient and the normal control to assess commonalities in RNA clonal evolution across the patients. We identified a total of 107 significantly (log2FC > 1 and q < 0.05) upregulated genes among the five patients (Additional file 4: Table S3). Venn diagram analysis showed that 8 of the genes overlapped with the 76 genes mentioned above: MIR222HG, ATP8B4, ADGRE2, ANKRD28, FLT3, PLAGL1, KDM5B, and CDK6. PCR analysis was performed on ANKRD28, CDK6, ADGRE2 and KDM5B and showed that these genes were significantly upregulated in AML (Fig. 6E–H). Similarly, survival analysis showed no significant significance.

**Fig. 7:**
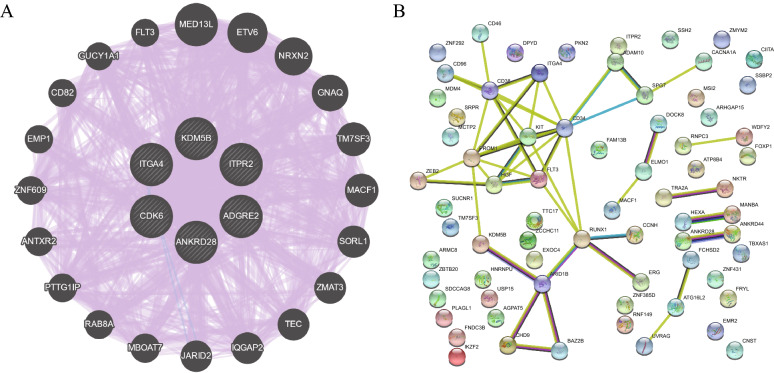
Gene–gene interaction network analysis of 6 target genes and protein‒protein interaction network analysis of the top 76 differentially expressed genes from cluster 12. **A** Gene‒gene interaction network. Nodes indicate genes, and lines indicate interactions. The purple line represents the gene coexpression network, and the green line represents the genetic interaction network. **B** Protein–protein interaction network. Nodes indicate proteins, and lines indicate interactions

To predict the functions of these six genes in AML, we performed protein‒protein and gene‒gene interaction network analyses (Fig. 7). There were 89.27% coexpression, 10.7% colocalization and 0.03% genetic interaction in the gene interaction network. As the target genes had no obvious correlations in the protein–protein interaction network, we performed protein‒protein interaction network analysis of the top 76 genes of cluster 12, which showed the highest associations between ITGA4 and AML-associated genes such as CD34, CD38, PROM1 and KIT.

## Discussion

At present, the treatment of NK-AML remains a challenge. The standard therapy for AML is still cytarabine- and anthracycline-based regimens (‘‘3 + 7’’ regimen) to achieve and maintain complete remission (CR) and cure AML. Approximately 60–80% of young people and 40–60% of elderly people (60 years or older) achieve initial remission after chemotherapy. However, at least 40% of these patients relapse with refractory disease, and the five-year survival rate is approximately 30% to 40% [20, 21]. LSCs are a key factor in cancer treatment failure and disease evolution. In AML, there are a small number of cells, including LSCs, side population cells and other stem cell-like cells, that possess the biological characteristics of quiescence, multilineage differentiation, self-renewal and disease maintenance, leading to disease relapse [22, 23]. Therefore, characterization of stem cell-like populations and the development of therapeutic strategies targeting these cells are the basis for achieving long-term remission of AML.

In this study, scRNA-seq was performed on BM mononuclear cells from 5 patients with NK-AML (M4/M5) and 1 normal control. The transcriptome profiles and gene expression patterns of the NK-AML (M4/M5) patients and healthy individual at the single-cell level were demonstrated. We captured a broad distribution of cell types, including monocytes, T cells, B cells, DCs, erythrocytes, and multiple leukemia cell populations. All 18 cell types were identified in all donors. Our results are consistent with the differential distribution of BM cells in healthy controls and AML patients and provide more evidence of the heterogeneity in NK-AML.

Due to cell scarcity, it is difficult to distinguish cancer stem cells (CSCs) using classic next-generation sequencing. In the scRNA-seq data, we found that cluster 12, which was rare in number, highly expressed not only multiple LSC markers, such as CD34, CD96 and CD133, but also the nontraditional LSC markers CD38 and CD46. CD38 is highly expressed mainly in multiple myeloma and chronic B-cell leukemia [24, 25]. CD46 is a key regulator of the classical and selective complement activation cascade in the innate immune system and is associated with a variety of immune inflammatory diseases [26]. GO and KEGG enrichment analyses showed that the DEGs of this cluster had biological functions related to the malignant pathways of AML. In AML, the first study of CSCs was published in the late twentieth century, in which Bonnet and Dick isolated a subset of CD34^+^/CD38^−^ leukemia cells. Compared with CD34^+^/CD38^+^ and CD34^−^ cells, CD34^+^/CD38^−^ cells can initiate AML in nonobese diabetic mice with severe combined immunodeficient disease (NOD/SCID mice) [27]. To date, the CD34^+^/CD38^−^ phenotype is still a recognized marker for LSC isolation. Our data showed that cluster 12 with high expression of CD34 and CD38 may be an LSC-like subpopulation, such as a side population. Hence, we speculated that the cluster might be an LSC-like group that plays important roles in NK-AML initiation and maintenance.

To explore gene expression patterns in this cluster, we identified 12 genes that were upregulated in AML and associated with prognosis in combination with data from the TCGA and GTEx databases. Studies on KIT, HGF and ERG in AML have demonstrated that these genes are all upregulated and that high expression predicts an unfavorable outcome [28–31]. Moreover, KIT mutations are common in AML. KIT is expressed on more than 10% of blasts in 64% of de novo AML cases and 95% of relapsed AML cases. Thus, KIT represents a potential therapeutic target in AML [32]. FCHSD2 is also highly expressed in AML, and its overexpression significantly increases cellular chemotherapy resistance. It was shown that FCHSD2 is a predictor of outcome in AML patients and that the determination of FCHSD2 expression at the time of diagnosis could help to predict the responses of AML patients to chemotherapy [33]. There are few studies on the DPYD, ARHGEF6, MCTP2 and SPN genes in AML, but these genes are known to be differentially expressed in other tumors, such as colorectal cancer, lung cancer and hepatocellular carcinoma. In addition, ITGA4 and ITPR2 were validated in AML datasets from the GEO database. Low expression levels of ITGA4 and ITPR2 indicated a poor outcome. The prognostic significance of ITPR2 was the opposite of the significance reported in the literature, which may be due to tissue differences and requires further validation in the future. Furthermore, the functional and prognostic significance of the other various genes requires future experimental clarification. PCR analysis further demonstrated that the expression levels of ITGA4, ITPR2, ADGRE2, ANKRD28, KDM5B, and CDK6 in NK-AML (M4/M5) were significantly higher than those in normal controls, suggesting that these genes may be possible biomarkers of NK-AML.

ITGA4, a member of the integrin alpha chain family of proteins, is considered to be an adverse prognostic factor for chronic lymphoblastic leukemia (CLL) with an invasive course and short time to treatment, and ITGA4 gene hypermethylation is a characteristic status in CLL compared with healthy controls [34, 35]^.^ Protein–protein interaction network analysis showed that ITGA4 was associated with PROM1, KIT, CD34 and CD38, which were closely related to AML. GO and KEGG enrichment analyses showed that ITGA4 was enriched in cell adhesion, leukocyte migration and hematopoietic processes and participated in the PI3K-Akt signaling pathway, which is related to tumor cell migration, adhesion, tumor angiogenesis and extracellular matrix degradation. ITPR2 is a key regulator of calcium ion transmembrane transportation and plays critical roles in cell migration, cell division, the cell cycle and proliferation. ANKRD28 is located at 3p25.1, and its function remains unclear. ANKRD28 is widely expressed in human tissues, especially in the BM, brain and testis. ITPR2 and ANKRD28 were demonstrated to be novel biomarkers for worse prognosis in NK-AML [36, 37]. ADGRE2 encodes a protein that is expressed mainly in myeloid cells and promotes cell‒cell adhesion. Upregulation of ADGRE2 was significantly associated with shorter OS in AML using publicly available genomic data [38]. KDM5B encodes a lysine-specific histone demethylase that belongs to the jumonji/ARID domain-containing family of histone demethylases. This protein plays a role in the transcriptional repression or certain tumor suppressor genes and is upregulated in certain cancer cells [39]. Downregulation of KDM5B produces efficient antileukemic effects in MLL-rearranged AML cells and HL-60 cells, suggesting that KDM5B may be a potential epigenetic target for AML treatment [40, 41]. CDK6 is a member of the CMGC family of serine/threonine protein kinases and is important for cell cycle regulation. CDK6 is highly expressed in AML patient samples and represents a promising target in MLL fusion-expressing, FLT3-ITD-positive and NUP98 fusion protein-driven AML [42, 43].

In summary, we leveraged the combination of single-cell transcriptomics and qRT-PCR to parse leukemia cells in NK-AML (M4/M5). Our results provide insight into the transcriptomic profiles of NK-AML (M4/M5) BM samples for the first time and identify a distinct LSC-like cell population with possible biomarkers in AML with a normal chromosomal karyotype. These findings provide a new population of LSCs and offer potential biomarkers and prognostic predictors for clinical applications in NK-AML. However, our experiment had a limited sample size, and the data may not be representative. In addition, the results have not been verified yet. Therefore, our next research step is to isolate the LSC-like cells according to their cell-surface markers identified by current data using flow cytometry. Quantitative Real-time PCR and Western blotting will be performed to detect the mRNA and protein expression of candidate marker genes to further verify our research results. The data and findings can guide therapeutic strategies aimed at malignant cells and target genes in NK-AML. Finally, the present study provides evidence that scRNA-seq plays an important role in the study of NK-AML and suggests that strategies promoting scRNA-seq may be valuable approaches for hematological malignancy therapy.

## Supplementary Information

**Additional file 1:Figure S1.** GO and KEGG pathway enrichment of cluster 0-cluster 17.

**Additional file 2:Table S1.** The basic informations and sequencing data of NK-AML (M4/M5) patients and healthy donors.

**Additional file 3:Table S2.** The top 76 up-regulated genes of cluster 12 (log2FC > 2).

**Additional file 4:Table S3.** The common genes significantly upregulated in five NK-AML(M4/M5) patients (log2FC > 1 and q < 0.05).

## Data Availability

The datasets used and/or analyzed during the current study are available from the corresponding author on reasonable request.
